# Psychological distress is more common in some occupations and increases with job tenure: a thirty-seven year panel study in the United States

**DOI:** 10.1186/s40359-023-01119-0

**Published:** 2023-03-31

**Authors:** James N. Laditka, Sarah B. Laditka, Ahmed A. Arif, Oluwaseun J. Adeyemi

**Affiliations:** 1 Department of Public Health Sciences, University of North Carolina at Charlotte, 9201 University City Boulevard, Charlotte, NC 28223 USA; 2 Department of Emergency Medicine, New York University Grossman School of Medicine, 550 1st Avenue, New York City, NY 10016 USA

**Keywords:** Anxiety, Depression, Mental health, Occupational health, Psychological distress

## Abstract

**Background:**

Workers in certain occupations may have elevated risks of psychological distress. However, research is limited. For example, researchers often measure distress that may have existed before occupational exposures. We studied occupations and the development of psychological distress using national data from the United States.

**Methods:**

We reviewed relevant research to identify occupations with low and high risks of mental health problems. We confirmed those individual low and high risk occupations using 1981–2017 data from the Panel Study of Income Dynamics (n = 24,789). We measured new cases of distress using the Screening Scale for Psychological Distress (Kessler K6) and compared distress in the low and high risk groups, adjusted for factors associated with occupational selection and non-occupational distress risks. A subset of participants described their jobs (n = 1,484), including factors such as job demands, social support, and control over work. We examined associations of those factors with psychological distress.

**Results:**

Workers in high risk occupations had 20% higher adjusted odds of developing distress than those in low risk occupations (odds ratio, OR 1.20, 95% confidence interval, CI 1.13–1.28). Distress increased with time in a high risk occupation: ≥5 years OR 1.38 (CI 1.18–1.62), ≥ 10 years OR 1.46 (CI 1.07–1.99), and ≥ 15 years OR 1.77 (CI 1.08–2.90; p-trend = 0.0145). The most common positive participant descriptions of their jobs indicated social support (34%), sense of accomplishment (17%), and control over work (15%). Participants reporting such descriptions were significantly less likely to have a high risk occupation (OR 0.66, CI 0.46–0.94, p = 0.0195). The most common negative descriptions were excessive job demands (43%), low social support (27%), and lack of control (14%). Participants reporting such descriptions were significantly more likely to have a high risk occupation (OR 1.49, CI 1.03–2.14, p = 0.0331).

**Conclusion:**

Certain occupations may have high risks of psychological distress, which may be due to characteristics of the occupations rather than employee characteristics, or in addition to them. Results were consistent with theoretical models of psychosocial work environments. Providers of health care and social services should ask patients or clients about work-related distress.

**Supplementary Information:**

The online version contains supplementary material available at 10.1186/s40359-023-01119-0.

## Introduction

Mental health disorders cause more work absence and work disability than musculoskeletal problems [1, 2]. In a given year about 19 million Americans have a major depressive episode [3]. Nearly half of them also have anxiety disorders [4, 5]. These disorders cost employers nearly $200 billion in 2018 [3]. Occupation-related mental health problems may be due to job strain when workers have high physical or psychological job demands yet little control over work processes [6], limited support at work, limited ability to use their skills or discretion in how they do so, low or inconsistent income, or low occupational prestige [7–10]. There may be many such factors [8], characteristics of the Job-Demand-Control-Support Model.

Meta-analyses have generally found that the Job-Demand-Control-Support Model predicts sickness absence due to diagnosed mental disorders [11], and depression [12, 13]. Mikkelsen et al. [14] reviewed 54 studies from developed countries of associations of psychosocial stressors at work with depressive disorders. They found evidence of such associations for low relational justice (e.g., employer treats workers unkindly or with bias, or does not: consider workers’ views or rights, deal with workers truthfully, or provide timely feedback), effort-reward imbalance, and job strain, with statistically significant summary risk ratios, respectively, 1.60, 1.53, and 1.14 [14]. However, the majority of studies had limited controls for confounding, including whether participants had depressive disorders at baseline [14]. Mikkelsen et al. [14] judged that the evidence was inadequate to conclude that psychosocial exposures at work “were either likely or unlikely” to cause depressive disorders (p. 479). None of the studies examined in these meta-analyses considered whether certain occupations may be more likely than others to expose workers to higher risks for developing mental health problems [14].

Psychological distress is a measure of mental and behavioral health, sometimes used to describe physiological and behavioral symptoms that are not specific to a given pathology, such as anxious and depressive reactions, irritability, impaired cognitive function, sleep disturbance, and work absenteeism [15]. Other researchers include specific mental or behavioral problems in psychological distress, such as diagnoses of anxiety or depression [4, 15–17]. Our study focuses on a widely used and extensively validated definition of psychological distress that encompasses both non-specific symptoms and specific diagnoses such as anxiety and depression [18–20]. We refer to occupations with elevated risks of developing psychological distress as high risk occupations. In contrast, researchers found that positive job resources (e.g., skill discretion, social support, and skill utilization) were associated with lower risks of depressive or anxiety symptoms [6–10]. We refer to occupations with low risks of developing psychological distress as low risk occupations.

Marchand, Demers, and Durand (2005) [21] cited 27 studies that identified characteristics of high risk occupations: repetitive work, low skill utilization and decision authority; physical challenges; psychological and emotional demands caused by workload, work pace, conflicting requests, and role ambiguity; irregular schedules and long hours; physical, sexual and psychological harassment; and performance pay. However, most of the studies were limited to cross-sectional data. Moreover, studies had mixed findings. For example, one study found a 20% higher prevalence of depression among health care workers, compared to all other occupations [22]. In another study, health care professionals had less psychological distress than other professionals [23].

Results of studies may often be due to selection effects [24, 25]. People with higher mental health risks may sort into certain occupations due to choices made by the workers or employers [8, 16, 26]. Addressing selection effects requires longitudinal data and controls for occupational opportunity and choice [16, 27, 28]. Few researchers address those needs [23].

People also face distress risks outside the workplace, yet researchers rarely control for non-occupational risks [16, 17, 22, 26, 29]. One prospective study in Canada found that factors outside the workplace were substantially associated with the development of psychological distress, and that occupation was not a significant factor when those characteristics were controlled [30].

In another area, a meta-analysis found that workers in high risk occupations for longer periods had higher mental health risks, although that study was limited to hospitalization for depression and two measurements of occupation about five years apart [12]. Many studies are limited to small samples, brief periods, individual occupations, or only one or a few states [9, 16, 17, 22, 26, 28]. Most do not distinguish recent mental health problems from longstanding conditions [22], or from those that precede occupational exposures[24, 25].

### Hypotheses

We studied associations of occupations with the development of psychological distress in the United States using nationally representative data across 37 years. We hypothesized that: (1) people in high risk occupations would be more likely than others to develop distress, and that these high risks would persist when adjusted for selection effects and non-occupational factors; (2) the risk of developing distress would be greater for people in high risk occupations for longer periods; and (3) workers’ descriptions of their jobs would be associated with characteristics of the Job-Demand-Control-Support Model, and linked with occupational risk of developing psychological distress.

## Methods

### Data

Data were from the Panel Study of Income Dynamics (PSID), which began in 1968 with about 18,000 participants in 5,000 households [31–35]. The PSID interviewed participants annually through 1997, then every 2 years. Response rates are 96–98%. The PSID maintains national representativeness [32, 33, 35]. Modest attrition has not biased national estimates [32, 33]. Health questions and results are similar to those of national health surveys [31].

### Measuring occupation

We measured occupation from 1981, when the PSID began measuring occupation in the manner used for this research, through 2017. The PSID asked three open-ended questions of household principal respondents and their spouses or partners: “What is your main occupation? What sort of work do you do?” and “Tell me a little more about what you do.” Based on the verbatim responses, PSID staff assigned 3-digit Census occupation codes, a process found reliable [36]. Data through 2001 used 435 3-digit 1970 Census codes; 148 defined lower and higher risk occupations (Supplemental Table 1). Data for later years used analogous year 2000 Census codes (Supplemental Table 2). Most of the occupations were defined as occupational groups. For example, 83 different codes identified various types of technicians in the year 2000 codes (Supplemental Table 2).

### Measuring distress

The analysis used multiple analytic observations for each participant. Each analytic observation represented the period between survey waves, typically two years, including occupation measured at the beginning of that period and a measure of the development of distress during that period. The PSID measured distress at each wave, 2001–2017, using the Screening Scale for Psychological Distress (Kessler K6). Interviewers asked, “How often in the past month did you feel:” “nervous?” “hopeless?” “restless or fidgety?” “everything was an effort?” “so sad nothing could cheer you up?” and “worthless?” Participants chose responses from “1. All the time” to “5. None of the time.” In the standard practice, we scored responses in that order from 4 to 0 points and summed the scores, with a potential range 0–24; 13 or higher indicated clinically significant nonspecific distress (Cronbach’s alpha = 0.89) [18]. The K6 was designed to screen for anxiety, depression, and closely related conditions [18]. Although anxiety, depression, and psychological distress are conceptually distinct, they often co-occur and are highly correlated. Reports that a doctor or other health care professional diagnosed anxiety or depression also indicated distress.

We accounted for distress earlier in participants’ lives using lifetime health histories. Beginning in 2005, the PSID asked if a doctor or other health professional had ever diagnosed “any emotional, nervous, or psychiatric problem.” Those responding “yes” were asked if the diagnosis was for a specific condition including anxiety or depression, and the age at diagnosis. Using that information and all previous K6 measurements we identified new cases of distress for each analytic observation. To focus on new cases, we excluded participants from the risk set (the analytic data representing individuals who were at-risk of developing distress following an occupational exposure) for all years after distress onset.

### Measuring occupational risk

We first identified occupation groups with low and high distress risks, based on a review of relevant research. On average, people in the low risk occupations enjoy higher suitability of their jobs with their education and interests, relatively high skill discretion, adequate salaries, substantial control over workplace characteristics, high latitude and authority over decisions, interesting and varied work, relatively standard and consistent work hours, healthy working conditions, and social support from co-workers and managers [7]. High risk occupations often conflict with those characteristics. Table 1 shows the occupations with low distress risk, and relevant studies. Table 2 lists that information for high risk occupations. In the 14 studies cited across Tables 1 and 2, the measured outcomes were common mental disorders, depression, occupational stress and mental strain, and psychological distress; Agerbo et al. (2010) [37] and Roberts et al. (2013) [38] studied suicide risk.

**Table 1:** Occupations with low risk of developing psychological distress and related mental health conditions^a^

Occupation	Citation	Odds ratio (CI), p-value
Accountants	Grosch & Murphy (1998) [29]	0.39 (0.31–0.49), p < 0.0001
Architects	Agerbo et al. (2010) [37]; Fan et al. (2012) [16]; Shockey et al. (2017) [26]	0.09 (0.02–0.39), p = 0.0010
Directors, administrators	Agerbo et al. (2010) [37]; Fan et al. (2012) [16]; Grosch & Murphy (1998) [29]; Shockey et al. (2017) [26]; Stansfield et al. (2011) [17]	0.52 (0.48–0.56), p < 0.0001
Electricians	Eaton et al. (1990) [47]; Fan et al. (2012) [16]	0.41 (0.17–0.99), p = 0.0491
Engineers	Cadieux & Marchand (2014) [23]; Fan et al. (2012) [16]	0.53 (0.45–0.63), p < 0.0001
Farmers, fishery, forestry	Cohidon, Imbernon, & Gorldberg (2009) [48]; Fan et al. (2012) [16]; Roche et al. (2016) [49]; Shockey et al. (2017) [26]; Wang & Rosenman (2018) [22]	0.50 (0.39–0.65), p < 0.0001
Health aides	Fan et al. (2012) [16] [reported higher depression]	0.41 (0.18–0.92), p = 0.0304
Lawyers	Cadieux & Marchand (2014) [22]; Fan et al. (2012) [16]; Grosch & Murphy (1998) [29]	0.12 (0.02–0.88), p = 0.0367
Librarians	Fan et al. (2012) [16]; Shockey et al. (2017) [26]	(not applicable)^b^
Medical doctors	Cadieux & Marchand (2014) [22]; Grosch & Murphy (1998) [29]; Shockey et al. (2017) [26]	0.50 (0.42–0.60), p < 0.0001
Nurses	Cadieux & Marchand (2014) [22]; Fan et al. (2012) [16]; Shockey et al. (2017) [26]	0.46 (0.38–0.55), p < 0.0001
Pharmacists	Cadieux & Marchand (2014) [22]; Grosch & Murphy (1998) [29]	0.14 (0.09–0.22), p < 0.0001
Plumbers and pipe fitters	Bültmann, et al. (2001) [50]	0.54 (0.17–1.76), p = 0.3102^b^
Police officers	Eaton et al. (1990) [47]; Grosch & Murphy (1998) [29]	0.82 (0.67–0.98), p = 0.0336
Sales workers	Agerbo et al. (2010) [37]; Shockey et al. (2017) [26]	0.84 (0.74–0.95), p = 0.0059
Teachers	Agerbo et al. (2010) [37]; Grosch & Murphy (1998) [29]; Shockey et al. (2017) [26]	0.34 (0.30–0.39), p < 0.0001
Technicians	Agerbo et al. (2010) [37]; Grosch & Murphy (1998) [29]	0.65 (0.55–0.77), p < 0.0001

^a^Data source: Panel Study of Income Dynamics (PSID), 2003–2017. Results of discrete-time hazard analysis adjusted for age and sampling design; the reference group for the analysis of each occupation was all other occupations; Agerbo et al. (2010) [37] studied occupations linked with suicide.

^b^The longitudinal record included no cases of distress among librarians, and only three among plumbers and pipe fitters.

CI = 95% confidence interval.

**Table 2:** Occupations with elevated risk of developing psychological distress and related mental health conditions^a^

Occupation	Citation	Odds ratio (CI), p-value
Carpenters and joiners	Agerbo et al. (2010) [37]; Grosch & Murphy (1998) [29]	1.55 (1.15–2.08), p = 0.0035
Coal miners and operatives	Matamala Pizarro & Fuenzalida (2021) [51]; Roberts et al. (2013) [38]	1.72 (1.54–1.92), p < 0.0001
Cooks	Agerbo et al. (2010) [37]; Fan et al. (2012) [16]; Shockey et al. (2017) [26]; Wang & Rosenman, (2018) [121]	1.96 (1.74–2.21), p < 0.0001
Laborers	Roberts et al. (2013) [38]; Grosch & Murphy (1998) [29]	1.61 (1.38–1.88), p < 0.0001
Personal services workers	Fan et al. (2012) [16]; Wang & Rosenman (2018) [22]	1.62 (1.26–2.06), p = 0.0001
Plant and machine assemblers	Agerbo et al. (2010) [37]; Fan et al. (2012) [16]	1.61 (1.14–2.27), p = 0.0065
Operatives except transport	Fan et al. (2012) [16]; Grosch & Murphy (1998) [29]; Mościcka-Teske et al., (2017) [52]	1.72 (1.43–2.06), p < 0.0001
Road construction workers	Roberts et al. (2013) [38]	1.76 (1.42–2.46), p = 0.0009
Scaffolders and riggers	Roberts et al. (2013) [38]	3.45 (1.70–7.01), p = 0.0006
Undertakers, funeral directors	Roberts et al. (2013) [38]	3.19 (2.16–4.73), p < 0.0001

^a^Data source: Panel Study of Income Dynamics (PSID), 2003–2017; discrete-time hazard analyses adjusted for age and sampling design; reference category was all other occupations; Agerbo et al. (2010) [37] and Roberts et al. (2013) [38] studied occupations linked with suicide. CI = 95% confidence interval.

We used age-adjusted discrete-time hazard analysis to examine each of the occupations shown in Tables 1 and 2, to confirm its risk status. Our decision rule required workers in each occupation selected as low or high risk to have statistically significant (p < 0.05) lower (or higher) odds of developing distress compared with the reference group of all other occupations. Tables 1 and 2 show the odds ratio (OR) and 95% confidence interval (CI) for each occupation group. For example, the high risk result for laborers was OR 1.92 (CI 1.62–2.28); the low risk result for lawyers was OR 0.12 (CI 0.02–0.88). We then created a variable that indicated whether the participant had an occupation hypothesized to have low distress risk, and another variable indicating whether the participant had an occupation hypothesized to have high distress risk.

### Participants’ descriptions of their jobs

A subset of participants described their jobs in 1972 (n = 1,484). The PSID asked how much participants enjoyed their jobs, and why they answered as they did. Verbatim responses were similar to the workplace characteristics of the Job-Demand-Control-Support Model. The PSID coded responses in six categories: social support (e.g., “I like the people I work with” or “I like my boss”), sense of accomplishment, control over work, manageable job demands, work congruent with the participant’s training, and a category indicating generally enjoyable work. Corresponding categories indicated reasons why participants disliked their work, such as lack of social support. Coders were trained and supervised by the head of the Coding Section at the University of Michigan Survey Research Center [39]. About 10% of the interviews were coded twice, by both the coder and an expert check-coder (sometimes called the “gold standard”). The twice-coded interviews indicated that coding error rates were less than 2% [39]. Given limited sample sizes we summarized the six categories with a dichotomous variable indicating positive or negative job descriptions.

### Control variables

Variables with fixed values across all analytic observations were: sex; race/ethnicity (African American, Hispanic, non-Hispanic white, or other); childhood health; and midlife obesity and physical activity. Variables updated for each analytic observation were age, education, income, rural residence, and measures of non-occupational stress: having a child with a developmental disability, a family member in poor health, limited family support (no spouse or partner), divorce or widowhood in the past three years, and any family member currently unemployed. We measured income as the ratio of household income to the Census needs standard, the income level that defines the poverty threshold, which is adjusted for family size, the number of children and older persons in the household, and the area cost of living [40]. Rurality indicated “completely rural” counties, where the reference category was all other area types. Completely rural counties have no population center with more than 2,500 residents and are not closely tied with an urban area economically.

### Occupation and job tenure

We examined three measures of occupational risk, each updated for each analytic observation: (1) current work in a high risk occupation; (2) number of years in a high risk occupation (job tenure); and (3) having at least 5, 10, or 15 years in a high risk occupation.

### Analysis

The baseline for measuring each individual’s occupational history and tenure in low or high risk occupations was 1981. The baseline distress measurement was 2001; the development of new cases of distress was measured in each survey wave thereafter, 2003–2017. Inclusion criteria were: ages 18–65 at any time between 1981 and 2017; occupation information and the K6 provided at least twice; nonmissing data for all covariates; and a positive PSID sampling weight. Weighted results represent adult residents of the United States not in institutions [33]. In addition to descriptive methods, we used logistic discrete-time hazard analysis adjusted for repeated measures with generalized estimating equations. To examine associations of workers’ positive or negative job descriptions with distress, we estimated logistic regressions that accounted for age, sex, and the sampling design.

We conducted the analyses using SAS 9.4 (Cary, North Carolina). All methods were carried out in accordance with relevant guidelines and regulations for research ethics. The University of Michigan Health Sciences and Behavioral Sciences Institutional Review Board conducts an annual review of the PSID data collection and distribution protocols and survey instruments to ensure the rights and welfare of research participants are protected. The PSID obtains informed consent from all participants. The Office of Research Compliance at the University of North Carolina at Charlotte determined that this analysis did not require Institutional Review Board approval. We did not use experimental protocols.

## Results

We examined occupation histories of 24,789 participants, with 204,159 analytic observations. The average number of years in high risk occupations was 6.7 (standard deviation, SD 5.3); 7.7% (SD 2.7) of the sample had at least 10 years in a high risk occupation, averaging 15.7 years (SD 4.5). The average number of K6 measurements per respondent was 5.1 (SD 0.8).

### Sample characteristics

Table 3 shows sample characteristics for 2001. The estimated weighted prevalence of K6 psychological distress was 6.8% (CI 5.8–7.8). Including diagnoses of anxiety or depression increased that estimate to 11.4% (CI 10.2–12.5). The PSID over-sampled African Americans, who were 31.1% of the unweighted sample.

**Table 3:** Sample characteristics at baseline^a^

	Unweighted			Weighted			
Measure	%	(SD)		%	LB	UB	
“Distress” (any of the following)	12.5			11.4	10.2	12.5	
Anxiety or depression diagnosis	3.7			4.8	4.1	5.4	
Psychological distress (K6)	8.6			6.8	5.8	7.8	
High risk occupation ≥ 10 years	7.7			9.0	8.0	10.0	
High risk occupation current	35.1			31.5	30.2	32.8	
Age in years, mean	43.4	(13.6)		40.1	39.6	40.6	
Rural resident	5.9			6.9	5.4	8.4	
Midlife obesity	18.5			15.5	14.2	16.9	
Midlife sedentary	13.8			6.8	5.8	7.7	
Education							
< High school	13.6			10.8	9.4	12.2	
GED	7.6			6.3	5.5	7.2	
High school diploma	61.7			48.1	46.3	49.9	
Associate’s degree	7.8			8.0	7.4	8.7	
Bachelor’s degree	18.8			21.5	19.8	23.3	
Master’s degree or higher	8.0			9.9	8.7	11.0	
Income-to-need ratio, mean	7.7	(24.0)		8.4	7.5	9.2	
African American	31.1			13.2	10.0	16.4	
Hispanic	3.6			5.8	4.4	7.2	
Race, other	5.1			3.6	2.9	4.3	
White	60.1			77.4	73.5	81.2	
Female	50.9			51.3	49.7	52.9	
Married or partner	51.9			53.5	51.3	55.6	
Widowed, past 3 years	5.4			4.6	3.8	5.5	
Divorced, past 3 years	4.1			6.6	5.7	7.5	
Child with developmental disability	1.4			1.6	1.3	2.0	
Family member in poor health	6.9			6.8	4.8	8.7	
Unemployment ≥ 1 month	6.1			5.2	4.3	6.1	
Childhood fair/poor health	8.2			8.2	7.4	9.1	

^a^Data source: Panel Study of Income Dynamics, 2003–2017 (n = 24,789); occupation measured 1981–2017. Weighted results adjusted for sampling design (PSID over-samples African Americans). SD = standard deviation (shown for continuous variables). LB, UB = lower and upper bounds of the 95% confidence interval. Income-to-need ratio = ratio of family income to Census needs standard (poverty threshold). K6 = Screening Scale for Psychological Distress (Kessler K6). The baseline for a given individual was the first year with a distress measurement, most often 2001.

### High risk occupations and distress

Table 4 shows three models predicting distress. Participants who currently worked in a high risk occupation had 20% higher odds of developing distress than those in low risk occupations (OR 1.20, CI 1.13–1.28, p < 0.0001). Each additional year in a high risk occupation increased the odds of developing distress by 5% (OR 1.05, CI 1.00-1.10, p < 0.05). Participants with at least 5 years of high risk exposure had 38% higher odds of developing distress than those in low risk occupations (OR 1.38, CI 1.18–1.62, p = 0.0101, not shown); corresponding results were OR 1.46 (CI 1.07–1.99, p = 0.0160) for 10 years, and OR 1.77 (CI 1.08–2.90, p = 0.0237, not shown) for 15 years (p-trend = 0.0145).

**Table 4:** Association of high risk occupations with the development of distressa

		Measure of Exposure to High Risk Occupation													
		Current High Risk Occupation					Number of Years in High Risk Occupation					Ten or More Years In High Risk Occupation			
Measure	OR		LB	UB	P		OR	LB	UB	P		OR	LB	UB	P
High risk occupation		1.20	1.13	1.28	< 0.0001		1.05	1.00	1.10	0.0499		1.46	1.07	1.99	0.0160
Age in years		1.01	1.00	1.03	0.1270		1.01	1.00	1.03	0.1509		1.01	0.99	1.03	0.2456
Rural resident		0.91	0.57	1.47	0.7024		0.91	0.56	1.47	0.6988		0.92	0.57	1.48	0.7275
Midlife obesity		1.40	1.06	1.85	0.0174		1.40	1.06	1.85	0.0178		1.42	1.08	1.88	0.0120
Midlife sedentary		1.12	0.75	1.67	0.5779		1.12	0.75	1.67	0.5785		1.12	0.76	1.67	0.5670
Education															
< High school		1.74	1.24	2.43	0.0012		1.74	1.25	2.43	0.0012		1.78	1.27	2.47	0.0007
GED		1.49	1.01	2.20	0.0424		1.50	1.02	2.21	0.0414		1.50	1.02	2.21	0.0410
High school diploma		1.31	0.89	1.93	0.1721		1.31	0.89	1.94	0.1704		1.33	0.90	1.96	0.1566
Associate’s degree		0.86	0.58	1.26	0.4381		0.85	0.58	1.25	0.4098		0.86	0.58	1.26	0.4269
Bachelor’s degree		0.68	0.38	1.22	0.1992		0.67	0.38	1.21	0.1864		0.67	0.37	1.21	0.1881
Master’s degree or higher		0.96	0.94	0.97	< 0.0001		0.96	0.94	0.97	< 0.0001		0.96	0.94	0.97	< 0.0001
Income-to-Need ratio (/10)		0.76	0.57	1.00	0.0499		0.76	0.58	1.01	0.0557		0.76	0.57	1.00	0.0473
African American		0.64	0.40	1.03	0.0678		0.64	0.40	1.03	0.0674		0.69	0.43	1.11	0.1309
Hispanic		0.79	0.38	1.65	0.5330		0.79	0.38	1.65	0.5371		0.81	0.39	1.70	0.5804
Race, other		1.57	1.22	2.03	0.0004		1.58	1.23	2.04	0.0004		1.61	1.25	2.07	0.0002
White		0.68	0.58	0.79	< 0.0001		0.70	0.60	0.83	< 0.0001		0.68	0.58	0.79	< 0.0001
Female		1.27	0.94	1.73	0.1188		1.30	0.96	1.77	0.0900		1.27	0.94	1.72	0.1234
Married or partner		1.19	0.78	1.81	0.4255		1.21	0.79	1.84	0.3840		1.19	0.78	1.82	0.4086
Widowed, past 3 years		2.39	1.70	3.38	< 0.0001		2.40	1.70	3.39	< 0.0001		2.47	1.75	3.47	< 0.0001
Divorced, past 3 years		1.46	1.09	1.94	0.0099		1.45	1.09	1.94	0.0103		1.47	1.11	1.96	0.0081
Child with developmental disability		1.47	1.16	1.86	0.0014		1.46	1.16	1.86	0.0016		1.46	1.15	1.85	0.0017
Family member in poor health		1.48	1.14	1.94	0.0038		1.54	1.18	2.01	0.0015		1.51	1.16	1.97	0.0024
Unemployment ≥ 1 month		1.20	1.13	1.28	< 0.0001		1.05	1.00	1.10	0.0499		1.46	1.07	1.99	0.0160
Childhood fair/poor health		1.01	1.00	1.03	0.1270		1.01	1.00	1.03	0.1509		1.01	0.99	1.03	0.2456

^a^Data source: Panel Study of Income Dynamics, 2003–2017 (n = 24,789). Results of discrete-time hazard analysis. OR = odds ratio; LB, UB = lower and upper bounds of the 95% confidence interval.

Results suggested greater distress risk for some control variables than for high risk occupations (e.g., the death of a spouse, having a family member in poor health). However, the distress risks of high risk occupations persisted after controlling for those risk factors, and the distress risk increased greatly after long tenure in a high risk occupation.

### Occupational risk and participants’ descriptions of their jobs

Not shown in a table, the most common positive participant descriptions of their jobs indicated social support (34%), sense of accomplishment (17%), control over work (15%), and manageable job demands (10%). Participants who reported such descriptions were significantly less likely to have a high risk occupation (OR 0.66, CI 0.46–0.94, p = 0.0195). The most common negative job descriptions were excessive job demands (physically demanding jobs or too much pressure, 43%), low social support (conflicts with co-workers or managers, no chance to meet people or make friends, 27%), lack of control (14%), and lack of a sense of accomplishment (8%). Participants who reported such descriptions were significantly more likely to have a high risk occupation (OR 1.49, CI 1.03–2.14, p = 0.0331).

## Discussion

We studied associations of occupation with distress in the United States using nationally representative longitudinal data with repeated measures of occupation and psychological distress. We extended research in this area by focusing on new cases of distress following occupational exposures. Consistent with our first hypothesis, people with high risk occupations were significantly more likely to develop distress than those with low risk occupations [7, 16, 17, 22, 26, 28]. Given the variables for which we controlled, these risks are not likely due to characteristics of the workers or to nonoccupational distress risks—or not due only to them. Our results differed from the conclusions of a longitudinal study in Canada [30], which found little evidence linking most workplace factors with distress after controlling for non-occupational distress risks. However, that study and those of other researchers [7–9] did find evidence of a protective effect of social support at work for workers’ mental health.

We found a significant dose-response relationship between the number of years in a high risk occupation and distress. Consistent with our second hypothesis the odds of developing distress increased 5% with each additional year. This result was consistent with a metaanalysis linking tenure in high risk occupations with hospitalization for depression [12].

Consistent with our third hypothesis, participants who described positive job characteristics linked with the Job-Demand-Control-Support Model were significantly less likely than others to have high risk occupations. Participants who described negative job characteristics were significantly more likely to have high risk occupations. These results were consistent with researchers’ findings linking positive job resources (e.g., skill discretion, social support, and skill utilization) with a lower likelihood of depressive or anxiety symptoms [6–10].

### Limitations and strengths

Occupations other than those that we studied might also have low or high risks for distress, particularly if researchers have not studied the distress risks of those occupations—in which case they would not be included in our analysis. Although we reviewed the relevant literature, conducting an exhaustive literature review or meta-analysis was beyond the scope of our study. Further, researchers have found mixed results for a number of occupations. For example, Wang and Rosenman (2018) [22] found that depression was higher among health care workers, whereas Cadieux and Marchand (2014) [23] found that psychological distress was significantly lower in health care professions. Mixed results across studies may be due to differences in study designs, data and controls, time periods, and locations. We acknowledge limited theory associating specific individual occupations with distress. The Job-Demand-Control-Support Model provided a relevant framework [11].

Consistent with many studies [e.g., 16, 17, 22, 26, 28], the PSID provided only limited measures of organizational characteristics that may be associated with distress. We could not distinguish risk factors of occupations (e.g., exposures to chemicals) from characteristics of employers and industries (e.g., those with large workforces). The data did not measure organizational factors that may contribute to variation in distress outcomes across firms within a given occupation, such as organizational culture, organizational structure (such as vertical, horizontal, or matrixed organizations), organizational life stage, characteristics of the organization’s leadership and decision-making, and worker access to organizational resources or job security. Participants lived throughout the United States; it is likely that those in any given occupation represented a range of employer and organizational characteristics.

The external environment can also affect the risk that workers will develop distress, as when a regional or national recession increases job loss, or when a period of economic inflation reduces real income, increases uncertainty, and strains relations between employers and workers. It is likely that this source of variation was addressed to some degree by the fact that the data spanned several decades and represented a variety of changing external environments, including economic cycles. That variety reduced the likelihood of bias that can occur when cross-sectional studies represent only a single data collection period and therefore may not represent organizations over time.

High risk occupations were generally blue collar jobs. Low risk occupations generally had higher social status, although that group included lower status occupations such as health aides. If the controls did not adequately adjust for workers’ socioeconomic characteristics the results could measure social stratification rather than occupational differences. However, controls included education, income, and health in childhood and midlife, all of which are linked with socioeconomic status. In general, research on the social gradient of health shows poorer self-rated health, more limited physical functioning, and more long sickness absence for people with blue collar jobs, compared to people with higher socio-economic status. However, the evidence regarding mental health is not so clear. White collar workers have reported higher psychological job demands, while blue collar workers reported higher physical demands [41]. Studies found a reverse gradient for mental health outcomes; people with lower socioeconomic status were less likely than those with higher status to experience stress and burnout symptoms [42], as well as a wide range of other psychiatric symptoms[43–46]. A longitudinal study of occupations and psychological distress found no evidence that blue collar workers had an elevated risk of distress [30].

The K6 cut-point of 13 identified serious mental illness with substantial impairment, meeting criteria for a Diagnosis and Statistical Manual IV disorder [19]. Distress below that cutpoint can also have serious health and economic impacts [20].

We measured occupational exposures based on participants’ reports of their principal occupation in each survey wave. Americans increasingly work in more than one occupation at a given time. It would be useful to study the impact of multiple occupational exposures on distress.

Our study also had several strengths: use of nationally representative panel data with extended follow-up, many measurements of occupation for most participants, repeated measures using a validated indicator of psychological distress, and the focus on the development of distress following occupational exposures. We controlled for many individual-level characteristics that may influence occupation options and workers’ choices to enter or remain in an occupation. The panel data allowed us to examine whether occupational distress risks increased with tenure in high risk occupations.

The extended follow-up may also be a limitation as the association of some occupations with distress might have changed across the study period. For example, in recent years it has been reported that many doctors, nurses, and teachers are dissatisfied with their jobs, particularly since the beginning of the COVID-19 pandemic. In the PSID from 1972, however, 92.6% of medical doctors, nurses, and teachers described their jobs as “very enjoyable” (29.6%), “mostly enjoyable” (48.2%), or “somewhat enjoyable” (14.8%). Also, in the period following the study years the unprecedented system shock caused by the COVID-19 pandemic may have altered links between some occupations and distress.

Linking high risk occupations and distress does not necessarily establish causation as other factors might contribute to distress. However, our use of an extended period of longitudinal data with many measurements of occupation and distress, our focus on the development of distress following occupational exposure, and the dose-response relationship of occupational exposure with distress that we found, all provide evidence consistent with causation.

### Implications for practice and research

The Total Worker Health model at The National Institute for Occupational Safety and Health and The U.S. Surgeon General’s Framework for Workplace Mental Health and Well-being recognize the importance of promoting emotional wellbeing at work. The need is substantial and may be growing. In a 2021 survey of U.S. adult workers, 76% reported at least one mental health symptom, 17% points higher than two years earlier; 84% identified workplace factors as causes of their mental health problems [53]. More research is needed to test psychosocial work stress models using longitudinal data that ascertain mental health problems validly, provide adequate control for potential confounding, and include the measures required to test theory-based analytical models. Further research in this area may help employers develop effective strategies to promote a healthy and productive workforce.

## Conclusion

The results suggest that workers in certain occupations have relatively high risks of psychological distress, and that those risks may be due to occupation characteristics rather than worker characteristics and non-occupational distress risks—or in addition to them. Distress can greatly affect productivity, workplace climate, physical health, and employee satisfaction and retention. Providers of health care and social services should ask patients or clients about work-related distress.

## Electronic supplementary material

Below is the link to the electronic supplementary material.

Supplementary Table 1. 1970 Census occupation codes used in this analysis (for years 1981 to 2001)

Supplementary Table 2. 2000 Census occupation codes used in this analysis (for years 2003 to 2015)

## Data Availability

The data used for this study are available from the Panel Study of Income Dynamics, http://psidonline.isr.umich.edu/default.aspx. The collection of data used in this study was partly supported by the National Institutes of Health under grant number R01 HD069609, and the National Science Foundation under award number 1,157,698. By registering for access to PSID public release data, the User agrees that they will not transfer PSID public data that has been downloaded from the website, including user-created data extracts, to any third parties.

## Abbreviations

PSID: Panel Study of Income Dynamics
OR: Odds ratio
CI: 95% Confidence interval
K6: Kessler K6 Screening Scale for Psychological Distress
SD: Standard deviation
LB: Lower bound of the 95% Confidence Interval
UB: Upper bound of the 95% Confidence Interval.

## References

[1] Harvey SB, Modini M, Joyce S, Milligan-Saville JS, Tan L, Mykletun A et al. (2017). Can work make you mentally ill? A systematic meta-review of work related risk factors for common mental health problems. *Occup Environ Med* 2017; 74(4): 301–310. doi: 10.1097/00043764-199802000-00012. 10.1136/oemed-2016-104015 28108676

[2] Whiteford HA, Degenhardt L, Rehm J, Baxter AJ, Farrari AJ, Erskine HS et al. (2013). Global burden of disease attributable to mental and substance use disorders: Findings from the Global Burden of Disease Study 2010. *Lancet* 2013; 382:1575–1586. doi: 10.1016/S0140-6736(13)61611-6. 10.1016/S0140-6736(13)61611-6 23993280

[3] Greenberg PE, Fournier AA, Sisitsky T, Simes M, Berman R, Koenigsberg SH (2021). The economic burden of adults with major depressive disorder in the United States (2010 and 2018). PharmacoEconomics.

[4] Beekman AT, de Beurs E, van Balkom AJ, Deeg DJ, van Dyck R, van Tilburg W (2000). Anxiety and depression in later life: co-occurrence and communality of risk factors. Am J Psychiatry.

[5] Bandelow B, Michaelis S (2015). Epidemiology of anxiety disorders in the 21st century. Dialogues Clin Neurosc.

[6] Karasek R (1979). Job demands, job decision latitude, and mental strain: implications for job redesign. Adm Sci Q.

[7] Battams S, Roche AM, Fischer JA, Lee NK, Cameron J, Kostadinov V (2014). Workplace risk factors for anxiety and depression in male-dominated industries: a systematic review. Health Psychol Behav Med.

[8] Chamoux A, Lambert C, Vilmant A, Lanhers C, Agiius Boutaleb M (2018). Occupational exposure factors for mental and behavioural disorders at work: the FOREC thesaurus. PLoS ONE.

[9] Griffin JM, Greiner BA, Stansfeld SA, Marmot M (2007). The effect of self-reported and observed job conditions on depression and anxiety symptoms: a comparison of theoretical models. J Occup Health Psychol.

[10] Jung M, Lim S, Chi S (2020). Impact of work environment and occupational stress on safety behavior of individual construction workers. Int J Environ Res Public Health.

[11] Duchaine CS, Aubé K, Gilbert-Ouimet M, Vézina M, Ndjaboué R, Massamba V (2020). Psychosocial stressors at work and the risk of sickness absence due to a diagnosed mental disorder: a systematic review and meta-analysis. JAMA Psychiat.

[12] Madsen IEH, Nyberg ST, Magnusson Hanson LL, Ferrie JE, Ahola K, Alfredsson L (2017). Job strain as a risk factor for clinical depression: systematic review and meta-analysis with additional individual participant data. Psychol Med.

[13] Rugulies R, Aust B, Madsen IE (2017). Effort-reward imbalance at work and risk of depressive disorders: a systematic review and meta-analysis of prospective cohort studies. Scand J Work Environ Health.

[14] Mikkelsen S, Coggon D, Andersen JH, Casey P, Flachs EM, Kolstad HA (2021). Are depressive disorders caused by psychosocial stressors at work? A systematic review with meta-analysis. Eur J Epidemiol.

[15] Dohrenwend B, Shrout PE, Egri G, Mendelsohn FS (1980). Nonspecific psychological distress and other dimensions of psychopathology. Measures for use in the general population. Arch Gen Psychiatry.

[16] Fan ZJ, Bonauto DK, Foley MP, Anderson NJ, Yragui NL, Silverstein BA (2012). Occupation and the prevalence of current depression and frequent mental distress, WA BRFSS 2006 and 2008. Am J Ind Med.

[17] Stansfeld SA, Rasul FR, Head J, Singleton N (2011). Occupation and mental health in a national UK Survey. Soc Psychiatry Psychiat Epidemiol.

[18] Kessler RC, Andrews G, Colpe LJ, Hiripi E, Mroczek DK, Normand SL (2002). Short screening scales to monitor population prevalences and trends in nonspecific psychological distress. Psychol Med.

[19] Lace JW, Merz ZC, Grant AF, Emmert NA, Zane KL, Handel PJ (2020). Validation of the K6 and its depression and anxiety subscales for detecting nonspecific psychological distress and need for treatment. Curr Psychol.

[20] Prochaska JJ, Sung H-Y, Max W, Shi Y, Ong M (2012). Validity study of the K6 scale as a measure of moderate mental distress based on mental health treatment need and utilization. Int J Methods Psychiatr Res.

[21] Marchand A, Demers A, Durand P (2005). Does work really cause distress? The contribution of occupational structure and work organization to the experience of psychological distress. Soc Sci Med.

[22] Wang L, Rosenman K (2018). Adverse health outcomes among industrial and occupational sectors in Michigan. Preven Chronic Dis.

[23] Cadieux N, Marchand A. Psychological distress in the workforce: A multilevel and longitudinal analysis of the case of regulated occupations in Canada. *BMC Public Health* 2014;14, 808 (2014). 10.1186/1471-2458-14-808 10.1186/1471-2458-14-808 PMC4132901 25099686

[24] Stansfeld SA, Head JM, Marmot MG (1998). Explaining social class differences in depression and well-being. Soc Psychiatry Psychiatr Epidemiol.

[25] Stansfeld SA, North FM, White I, Marmot MG (1995). Work characteristics and psychiatric disorder in civil servants in London. J Epidemiol Community Health.

[26] Shockey TM, Zack M, Sussell A (2017). Health-related quality of life among US workers: variability across occupation groups. Am J Public Health.

[27] Strine T, Balluz L, Moriarty D, Mokdad A (2008). The associations between life satisfaction and health-related quality of life, chronic illness, and health behaviours among U.S. community-dwelling adults. J Community Health.

[28] Strine TW, Mokdad AH, Balluz LS, Gonzalez O, Crider R, Berry JT (2008). Depression and anxiety in the United States: findings from the 2006 behavioral risk factor Surveillance System. Psychiatr Serv.

[29] Grosch JW, Murphy LR (1998). Occupational differences in depression and global health: results from a national sample of US workers. J Occup Environ Med.

[30] Marchand A, Blanc ME (2010). The contribution of work and non-work factors to the onset of psychological distress: an eight-year prospective study of a representative sample of employees in Canada. J Occup Health.

[31] Andreski P, McGonagle K, Schoeni R, Institute for Social Research University of Michigan. An analysis of the quality of the health data in the Panel Study of Income Dynamics. Survey Research Center, 2009. Technical series paper #09 – 02. Available: https://psidonline.isr.umich.edu/publications/Papers/tsp/2009-02_Quality_Health_Data_PSID_.pdf Accessed 26 December 2020.

[32] Fitzgerald JM. Attrition in models of intergenerational links using the PSID with extensions to health and to sibling models. *B E J Econom Anal Policy* 2011; 11:pii. doi:10.2202/1935-1682.2868 10.2202/1935-1682.2868 PMC3285469 22368743

[33] Johnson DS, McGonagle KA, Freedman VA, Sastry N (2018). Fifty years of the Panel Study of Income Dynamics: Past, present, and future. Ann Am Acad Pol Soc Sci.

[34] Laditka JN, Laditka SB, Arif AA, Hoyle JN (2020). Work-related asthma in the USA: nationally representative estimates with extended follow-up. Occup Environ Med.

[35] Schoeni R, Stafford F, McGonagle KA, Andreski P (2013). Response rates in national surveys. Ann Am Acad Pol Soc Sci.

[36] Panel Study of Income Dynamics. A Panel Study of Income Dynamics: Procedures and tape codes, 1981 interviewing year, wave XIV, a supplement. Ann Arbor: Survey Research Center, Institute for Social Research, The University of Michigan. ; 1982. Available: https://psidonline.isr.umich.edu/data/Documentation/Fam/1981/81.PDF. Accessed 1 January 2021.

[37] Agerbo E, Gunnell D, Bonde JP, Mortensen PB, Nordentoft M (2007). Suicide and occupation: the impact of socio-economic, demographic and psychiatric differences. Psychol Med.

[38] Roberts SE, Jaremin B, Lloyd K (2013). High-risk occupations for suicide. Psychol Med.

[39] Survey Research Center. A Panel Study of Income Dynamics., 1968–1972 Interviewing years (Waves I-V), Volume 1, Study design, procedures, available data. Institute for Social Research, University of Michigan. Ann Arbor, MI, 1972. Available https://psidonline.isr.umich.edu/Data/Documentation/pdf_doc/psid68_72w1_5v1.pdf. Accessed 15 December 2022.

[40] National Research Council. (1995). Measuring poverty: A new approach. Washington, DC: The National Academies Press. Available: 10.17226/4759. [Accessed 2 January 2021].

[41] Schreuder KJ, Roelen CA, Koopmans PC, Groothoff JW (2008). Job demands and health complaints in white and blue collar workers. Work (Reading Mass).

[42] Hämmig O, Bauer GF (2013). The social gradient in work and health: a cross-sectional study exploring the relationship between working conditions and health inequalities. BMC Public Health.

[43] Dohrenwend BP (1990). Socioeconomic status (SES) and psychiatric disorders. Soc Psychiatry Psychiatr Epidemiol.

[44] Hudson CG (2005). Socioeconomic status and mental illness: tests of the social causation and selection hypotheses. Am J Orthopsychiatry.

[45] Johnson JG, Cohen P, Dohrenwend BP, Link BG, Brook JS (1990). A longitudinal investigation of social causation and social selection processes involved in the association between socioeconomic status and psychiatric disorders. J Abnorm Psychol.

[46] Lorant V, Deliège D, Eaton W, Robert A, Philippot P, Ansseau M (2003). Socioeconomic inequalities in depression: a meta-analysis. Am J Epidemiol.

[47] Eaton WW, Anthony JC, Mandel W, Garrison R (1990). Occupations and the prevalence of major depressive disorder. J Occup Med.

[48] Cohidon C, Imbernon E, Gorldberg M (2009). Prevalence of common mental disorders and their work consequences in France, according to occupational category. Am J Ind Med.

[49] Roche AM, Pidd K, Fischer JA, Lee N, Scarfe A, Kostadinov V (2016). Men, work, and mental health: a systematic review of depression in male-dominated industries and occupations. Saf Health Work.

[50] Bültmann U, Kant I, van Amelsvoort L, van den Brandt P, Kasl S (2001). Differences in fatigue and psychological distress across occupations: results from the Maastricht Cohort study of fatigue at work. J Occup Environ Med.

[51] Matamala Pizarro J, Aguayo Fuenzalida F (2021). Mental health in mine workers: a literature review. Ind Health.

[52] Mościcka-Teske A, Sadłowska-Wrzesińska J, Butlewski M, Misztal A, Jacukowicz A (2017). Stressful work characteristics, health indicators and work behavior: the case of machine operators. Int J Occup Saf Ergon.

[53] Mind Share P. 2021 Mental health at work report—the stakes have been raised. 2021. https://www.mindsharepartners.org/mentalhealthatworkreport-2021. Accessed 4 February 2023.

