# Supplementary material for: Psychological distress is more common in some occupations and increases with job tenure: a thirty-seven year panel study in the United States
Source: BMC Psychol. 2023 Mar 31;11:95. doi: 10.1186/s40359-023-01119-0 (PMC10064628; doi:10.1186/s40359-023-01119-0)
Supplement: Supplementary file 1 — Supplementary Table 1. 1970 Census occupation codes used in this analysis (for years 1981 to 2001) [file 40359_2023_1119_MOESM1_ESM.doc]

| Supplemental Table 1. 1970 Census occupation codes used in this analysis (for years 1981 to 2001) | |
| --- | --- |
| Occupation | Census codes |
| Accountants | 001 |
| Architects | 002 |
| Carpenters, joiners | 413 415 416 750 |
| Coal miners & operatives | 640 (where industry code 048) |
| Cooks | 912 |
| Directors, administrators,  officials, supervisors | 196 201 202 212 213 215 216 220 221 222 223 224 225 231 233 235 240 245 246 |
| Electricians | 430 431 433 |
| Engineers | 006 010 011 012 013 014 015 020 021 022 023 |
| Farmers, fishery, forestry | 025 752 801 806 |
| Health aides | 921 922 925 926 |
| Laborers | 780 785 796 822 823 824 |
| Lawyers | 030 031 |
| Librarians | 032 033 |
| Medical doctors | 062 063 065 071 |
| Nurses | 075 |
| Painters, plasterers | 510 511 520 521 |
| Personal care workers | 935 941 942 944 950 |
| Pharmacists | 064 |
| Plant & machine assemblers | 602 |
| Plant & machine operators | 461 462 650 651 652 653 656 690 692 694 695 |
| Plumbers, pipe ﬁtters | 522 523 |
| Protective services | 961 962 963 964 965 |
| Road construction | 436 |
| Sales workers | 260 262 265 270 271 280 296 |
| Scaﬀolders, riggers | 423 |
| Teachers | 102 103 104 105 110 111 112 113 114 115 116 120 121 122 123 124 125 126 128 130 131 132 133 134 135 140 141 142 143 145 |
| Technicians | 150 151 152 153 154 156 161 162 163 164 170 171 172 173 |
| Undertakers | 165 211 |
