# Supplementary material for: Psychological distress is more common in some occupations and increases with job tenure: a thirty-seven year panel study in the United States
Source: BMC Psychol. 2023 Mar 31;11:95. doi: 10.1186/s40359-023-01119-0 (PMC10064628; doi:10.1186/s40359-023-01119-0)
Supplement: Supplementary file 2 — Supplementary Table 2. 2000 Census occupation codes used in this analysis (for years 2003 to 2015) [file 40359_2023_1119_MOESM2_ESM.doc]

| Supplemental Table 2. 2000 Census occupation codes used in this analysis (for years 2003 to 2015) |
| --- |
| Occupation and Census codes |
| Accountants: 080 |
| Architects: 130 |
| Carpenters, joiners: 623 775 832 850 883 |
| Coal miners & operatives: 563 683 684 691 694 863 864 865 874 896 920 951 956 962 975 |
| Cooks: 402 403 405 780 785 |
| Directors, administrators, officials: 002 005 006 010 011 013 015 023 023 031 032 034 035 036 041 042 043 062 073 090 095 101 184 186 200 202 205 206 220 231 232 233 234 240 243 244 255 263 271 272 275 292 373 462 470 471 001 002 006 012 014 016 020 022 023 035 041 041 042 043 051 062 073 081 084 091 100 101 102 131 135 141 143 150 152 155 164 172 174 190 192 193 200 201 202 205 215 220 231 232 234 243 254 255 260 263 271 275 290 292 303 313 330 370 371 372 373 380 392 401 420 421 424 430 432 443 460 462 464 470 471 500 531 562 600 601 611 620 674 700 770 781 830 835 900 904 912 914 924 931 941 975 |
| Electricians: 635 703 741 711 772 |
| Engineers: 016 030 056 070 082 095 100 101 102 104 106 111 121 123 124 131 132 133 134 135 136 140 141 142 143 144 145 146 150 151 152 153 154 155 170 171 174 181 182 184 193 284 290 292 372 374 422 493 560 631 632 701 702 712 720 733 736 743 770 806 854 861 871 896 903 920 920 926 933 935 941 952 956 965 972 |
| Farmers, fishery, forestry: 020 021 600 605 610 612 |
| Health aides: 360 324 365 |
| Laborers: 422 423 425 435 605 612 613 626 674 694 761 896 930 935 936 926 962 975 |
| Lawyers: 210 |
| Librarians: 243 |
| Medical doctors: 300 306 312 |
| Nurses: 313 350 |
| Painters, plasterers: 642 646 660 881 |
| Personal care: 360 081 085 263 360 363 450 451 451 452 461 462 484 496 570 775 |
| Pharmacists: 165 172 305 |
| Plant & machine assemblers: 051 403 621 623 652 653 676 704 722 736 771 772 773 774 775 813 830 832 855 884 962 964 |
| Plant, machine operators: 193 290 414 443 503 511 580 581 582 585 590 592 605 613 624 625 626 630 632 636 660 675 680 682 684 691 693 694 761 773 775 781 783 784 785 792 793 794 795 796 800 801 802 804 810 812 814 815 820 822 823 825 826 830 832 834 836 840 841 842 843 846 853 854 860 861 862 863 864 865 871 872 873 874 880 881 883 884 885 886 890 893 894 895 896 913 920 950 951 952 953 956 960 961 963 965 974 975 |
| Plumbers, pipe ﬁtters: 644 |
| Protective services: 371 380 382 385 386 |
| Road construction: 436 613 630 632 626 673 881 |
| Sales: 005 051 052 071 086 263 282 406 470 471 472 474 475 476 480 481 482 484 485 490 492 494 495 496 512 521 524 535 541 592 913 913 936 |
| Scaﬀolders, riggers: 623 |
| Teachers: 220 |
| Technicians: 062 080 100 101 104 124 155 156 190 191 192 193 194 196 200 202 215 240 243 244 286 290 313 320 330 332 340 341 351 353 354 360 361 362 365 374 385 424 440 452 465 562 582 590 592 593 635 652 680 683 701 702 702 703 704 710 712 713 714 720 726 730 731 732 736 742 743 761 762 800 803 813 816 825 862 874 875 876 881 883 892 904 942 |
| Undertakers: 032 165 446 451 |
